# Meta-analysis of ART outcomes in women with different preconception TSH levels

**DOI:** 10.1186/s12958-018-0424-0

**Published:** 2018-11-05

**Authors:** T. Zhao, B. M. Chen, X. M. Zhao, Z. Y. Shan

**Affiliations:** 1 Department of Endocrinology and Metabolism, Institute of Endocrinology, First Affiliated Hospital, China Medical University, Shenyang, Liaoning China; 2 The First Affiliated Hospital of Sun Yat-sen University, Guangzhou, China; 3 Chengde Medical University, Chengde, Hebei China

**Keywords:** Thyroid-stimulating hormone, Pregnancy, Clinical pregnancy, Live birth, Miscarriage, Assisted reproduction technology

## Abstract

**Background:**

To assess whether elevated thyroid-stimulating hormone (TSH) levels before conception can predict poor outcomes of assisted reproductive technology (ART).

**Methods:**

Prior to July 2018, we searched the PubMed, EMBASE, COCHRANE, Google Scholar, and CNKI databases for studies. Retrospective or prospective reports that compared ART results in patients with subclinical hypothyroidism (SCH) with normal thyroid function were selected. Two reviewers separately reviewed each potential article for qualification, analyzed the quality of the studies according to the Newcastle-Ottawa scale, and extracted the data. The PRISMA guidelines were adopted.

**Results:**

We selected a total of 18 publications that included 14,846 participants for this meta-analysis. When the TSH cut-off value for SCH was set at 2.5 mIU/L, no significant differences were observed in ART-related outcomes between SCH patients and normal women. The evaluated outcomes included the live birth rate (LBR) (OR: 0.93; 95% CI (0.77,1.12), *P* = 0.43), clinical pregnancy rate (CPR) (OR:1.02; 95% CI (0.90,1.17); *P* = 0.74), pregnancy rate (PR) (OR: 1.00; 95% CI (0.89,1.12); *P* = 0.99), and miscarriage rate (MR) (OR:1.24; 95% CI (0.85, 1.80); *P* = 0.26). Furthermore, when a higher TSH level was used as the cut-off value to diagnose SCH (i.e., 3.5–5 mIU/L), a significant difference was found in the MR (OR: 1.91; 95% CI (1.09, 3.35); *P* = 0.02) between the two groups of ART-treated women. However, when a broader cut-off value was used to define SCH, no significant differences were observed in the LBR (OR: 0.72; 95% CI (0.47,1.11); *P* = 0.14), CPR (OR: 0.82; 95% CI (0.66,1.00); *P* = 0.052), or PR (OR: 1.07; 95% CI (0.72,1.60); *P* = 0.74) between the two groups of ART-treated women.

**Conclusion:**

No difference was observed in ART outcomes when a TSH cut-off value of 2.5 mIU/L was used. However, when a broader TSH cut-off value was used, preconception SCH resulted in a higher miscarriage rate than in normal women.

**Electronic supplementary material:**

The online version of this article (10.1186/s12958-018-0424-0) contains supplementary material, which is available to authorized users.

## Background

SCH affects 2–5% of all pregnant women in the United States. This disease is characterized by abnormalities in the hypothalamic-pituitary-thyroid axis that are reflected by alter standard serum thyroxine (T4) levels and increased serum TSH levels [1]. Opinions vary regarding the different guidelines available related to the treatment of women with SCH seeking to use ART because the optimal therapy for these patients remains unknown. This is partly because of limitations related to a lack of consequence data on miscarriages that occur in early pregnancy.

Overt hypothyroidism (OH) is defined as a high level of TSH and a low level of T4 and is related to infertility as well as adverse pregnancy outcomes [2], including early pregnancy miscarriage, stillbirth and preterm delivery [3–5]. Recently, many studies have emerged that have evaluated the association between SCH during pregnancy and multiple poor maternal outcomes [6–8]. However, their conclusions have not been consistent. One meta-analysis demonstrated that SCH during pregnancy is related to multiple adverse neonatal and maternal consequences [9].

The relationship between ART outcomes and thyroid function has been a hot topic and the subject of a great deal of debate in recent years [10]. The reason for this interest seems obvious, at least from an epidemiological point of view, because ART is continually being performed and thyroid disorders are highly prevalent in women of reproductive age. In accordance with the results described in a recent report by the European Society of Human Reproduction and Embryology (ESHRE), between 0.8 and 4.1% of children born in Europe are the result of in vitro fertilization (IVF) [11]. Until recently, societies governing the use of human reproductive technologies did not recommend measuring TSH levels in asymptomatic ovulatory women [12, 13]. However, the new guidelines reported by the American Society for Reproductive Medicine (ASRM) endorse measuring TSH concentrations in infertile women seeking to become pregnant [14]. The prevalence of thyroid disorders has increased in subfertile women, and this has been recognized by the American Thyroid Association (ATA) [15] and the Endocrine Society. These organizations have consequently produced recommendations regarding the measurement of TSH levels in woman at “high-risk” of thyroid disease, including asymptomatic infertile patients [16].

During a pregnancy, variations occur in the normal range of TSH levels, and this has led to the introduction of SCH, a more vaguely defined complication observed in pregnant patients [17]. While the lower range of normal serum TSH levels has remained consistent, the upper limit for normal TSH levels has changed dramatically in recent years [18]. The ATA and The National Association of Clinical Biochemistry (NACB) [19] have lowered the upper limit for normal TSH levels in the first trimester of pregnancy from 4.5 mIU/L to 2.5 mIU/L. [15] Other clinical institutions, such as the American Association for Clinical Chemistry (AACC), have traditionally set 5.0 mIU/L as the upper limit for normal TSH levels [20].

ART patients experience many barriers when attempting to achieve conception, and identifying the optimal range of pregestational TSH levels is now specifically recognized as an important parameter. Furthermore, the superovulation caused by ART generates a rapid increase in E2 levels [21] that increases the hepatic synthesis of thyroid-binding globulin and leads to a reduction in free, unbound T4 [22–24]. These events can potentially aggravate the condition of a patient with underlying SCH. The risk of poor iatrogenic-related reproductive outcomes in ART has made SCH a special focus in the ART population.

Though obstetricians can correct TSH in patients who are already pregnant, by inspecting data associated with a large cohort of ART patients, in this study, we attempt to determine whether elevated TSH levels in the preconception period predict adverse outcomes in ART patients. We seek to address whether it is appropriate to apply traditional TSH criteria in patients about to undergo ART (i.e., are these criteria associated with any clinical benefit) and to prompt reproductive medical organizations to develop methods to increase the ability of practitioners to identify and treat ART patients whose TSH status puts them at high risk of adverse results.

## Methods

### Eligibility criteria

The included studies were limited to prospective or retrospective studies that compared ART consequences in patients with SCH with normal thyroid function. Publications were excluded if (1) only women with SCH were described without a comparison group consisting of women without SCH, (2) the included women had overt hypothyroidism or hyperthyroidism (3) TSH was not evaluated before the ovarian stimulation cycle we begun, (4) the number of IVF/ICSI cycles was not specified, (5) no specific TSH cut-off value was used to define SCH.

### Information sources and search

These searches were performed in the Medline, PubMed, EMBASE and COCHRANE, Google Scholar, CNKI databases. For instance, Medline was searched using the following search string for articles published from January 1990 to July 2018: (((“insemination”[MeSH Terms] OR “insemination”[All Fields]) OR (“fertilization in vitro”[All Fields] OR “fertilization in vitro”[MeSH Terms] OR (“fertilization”[All Fields] AND “vitro”[All Fields]) OR “fertilization in vitro”[All Fields])) OR (“reproductive techniques”[MeSH Terms] OR (“reproductive”[All Fields] AND “techniques”[All Fields]) OR “reproductive techniques”[All Fields])) AND ((((“hypothyroidism”[MeSH Terms] OR “hypothyroidism”[All Fields]) OR ((“thyroid gland”[MeSH Terms] OR (“thyroid”[All Fields] AND “gland”[All Fields]) OR “thyroid gland”[All Fields] OR “thyroid”[All Fields] OR “thyroid (usp)”[MeSH Terms] OR (“thyroid”[All Fields] AND “(usp)”[All Fields]) OR “thyroid (usp)”[All Fields]) AND (“physiopathology”[Subheading] OR “physiopathology”[All Fields] OR “dysfunction”[All Fields]))) OR (“thyroid diseases”[MeSH Terms] OR (“thyroid”[All Fields] AND “diseases”[All Fields]) OR “thyroid diseases”[All Fields] OR (“thyroid”[All Fields] AND “disorder”[All Fields]) OR “thyroid disorder”[All Fields])) OR (“thyroid diseases”[MeSH Terms] OR (“thyroid”[All Fields] AND “diseases”[All Fields]) OR “thyroid diseases”[All Fields] OR (“thyroid”[All Fields] AND “disease”[All Fields]) OR “thyroid disease”[All Fields])). We included all published retrospective or prospective studies. All relevant publications were retrieved. We also systematically reviewed the reference lists of the identified articles to identify additional reports that could be included in the meta-analysis. We made no attempt to identify unpublished reports.

Study selection and Data Collection Processes.

**Fig. 1:**
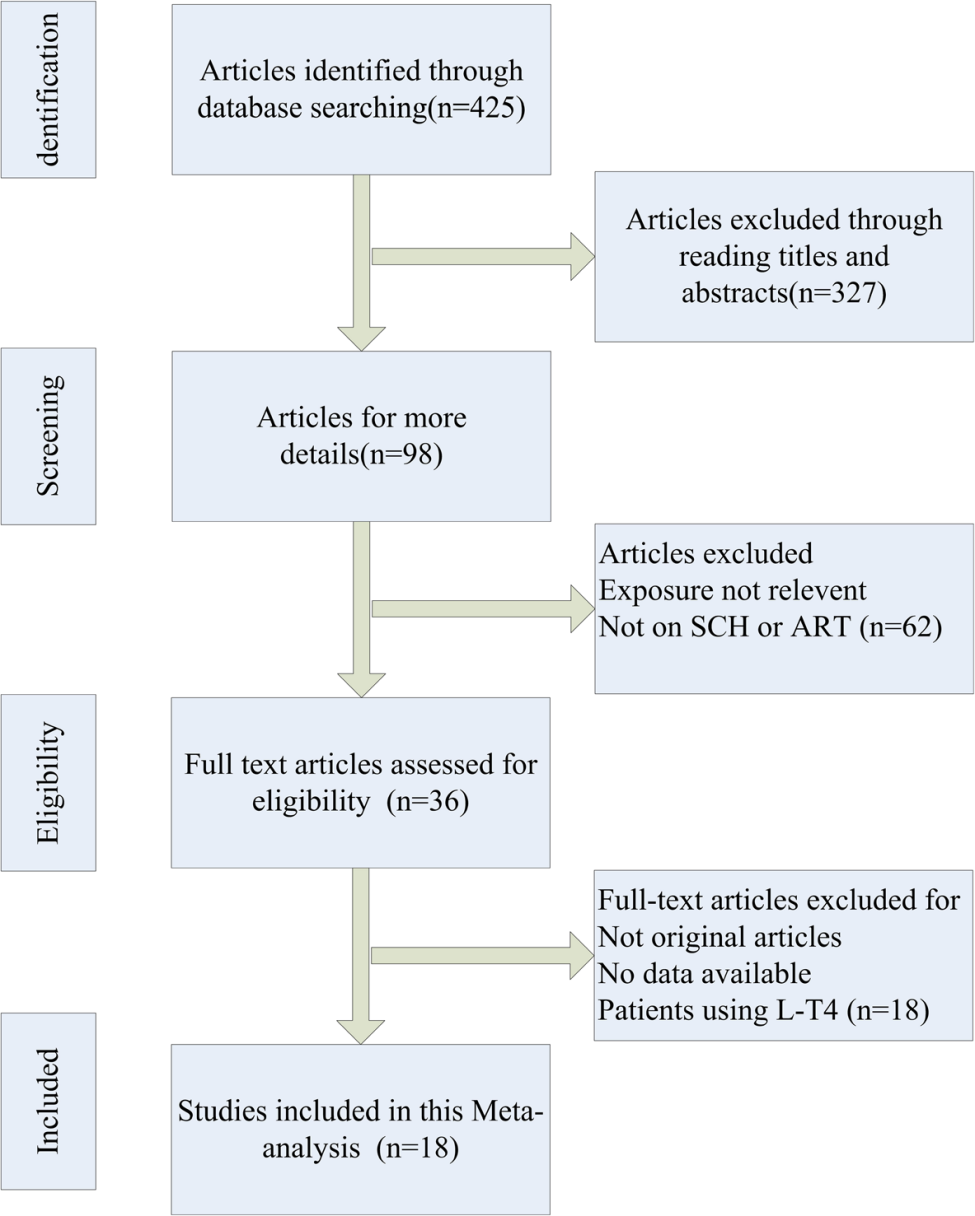
Process of study selection. SCH, subclinical hypothyroidism. ART, assisted reproduction technology. L-T4, levothyroxine

Two authors (Zhao T and Chen BM) performed the original screening of the titles and abstracts of all studies, and citations considered irrelevant by both observers were excluded. The PRISMA flow diagram (Fig. 1) provided more detailed information on the selection process of articles. Two authors (Zhao T and Chen BM) independently extracted all research data into normative forms. When there was a difference of opinion, the third author (Zhao XM) consulted with the two authors to achieve consensus. The year of publication, country, setting, study design, number of participants, clinical characteristics of the study subjects, thyroid function assays used, and ART (IVF, ICSI or intrauterine insemination (IUI)) implemented in the comparable groups were recorded.

### Quality assessment

We evaluated the quality of the articles according to the Newcastle–Ottawa scale, which is an effective method for scoring observational and non-randomized studies. All articles were evaluated independently by two authors. Discrepancies were solved by consensus. The Newcastle–Ottawa scale employs a score system based on the following three primary criteria: the selection of participants, the comparability of study groups, and outcome or, for case-control studies, an assessment of exposure. While ‘comparability of cohorts’ was scored as 2, 1 or 0; the other two primary criteria were scored based on eight items, each scored as either 0 or 1. Accordingly, the quantitative estimation of the total quality of each individual article ranged from 0 to 9. In the meta-analysis, articles were considered high quality if they obtained seven or more scores, those obtaining four to six scores were believed medium quality, and those obtaining three or less scores were believed low quality.

### Data items

We extracted information related to the research characteristics, the quality of the publications and the test results from each included article. The primary outcome was the LBR per woman, which was defined as the number of childbirths that led to one live born baby. The following secondary outcomes were applied: (i) the CPR per patient, which was defined using conceptions diagnosed by ultrasonography, including cases in which there was one or more gestational sacs in the uterus; (ii) the PR per woman, with pregnancies diagnosed as serum b-hCG levels ≥5 mIU/mL within 14 days following ART; and (iii) the MR per clinical conception.

### Risk of bias

We adopted the Cochrane Collaboration’s tool for assessing risk of bias to evaluate the risk of bias in individual studies. We analyzed the following risk of biases: 1) selection bias (i.e., bias introduced by the selection of individuals, groups or data for analysis in such a way that proper randomization is not achieved, thereby ensuring that the sample obtained is not representative of the population intended to be analyzed) 2) performance bias (i.e., bias due to the knowledge of the allocated interventions by personnel and participants during the research) 3) detection bias (i.e., bias due to randomization of the allocated interventions is not achieved by outcome assessors) 4) attrition bias (i.e., bias due to loss of follow-up, withdrawal, and no response during the study) 5) reporting bias (i.e., selective revealing or suppression of the outcomes).

Except for the above-mentioned types of biases, other two types of biases were included 1) sampling bias (i.e., bias leading to samples can not represent all the study population, mainly associated with the subject selection problem which can undermine the generalization of outcomes) 2) measurement bias (i.e., bias resulting from inappropriate use of tests or scales to measurement of preconception TSH values and ART outcomes mainly related to inconsistent or non-validated criteria).

### Summary measures and synthesis of results

We used Stata (version 11) to analyze the data. We calculated a combined odds ratio (OR) with a 95% confidence interval (CI) to assess the strength of the connection between SCH and the risk of adverse pregnancy-related outcomes. The significance of the combined OR (counted using the Mantel–Haenszel statistical approach) was identified with the Z test. We defined significance as a *P* value less than 0.05. Random effects and fixed effects models were used in this meta-analysis. To evaluate between-study heterogeneity, both the ×2-based Q statistic test and the I2 statistic were applied. We defined I2 values of 25%, 50%, and 75% as representing low, moderate, and high heterogeneity, respectively. We used a random effects model to pool outcomes when high heterogeneity was observed; whereas we used a fixed effects model to pool outcomes when heterogeneity was not high.

### Additional analyses

We evaluated the effect of each article on the overall risk appraisal by sequentially omitting each report to validate the authenticity of the results of the meta-analysis. We utilized Begg’s funnel plots and Egger’s linear regression test to estimate potential publication bias. The present study fulfilled the criteria of Preferred Reporting Items for Systematic reviews and Meta-Analyses (PRISMA) (Additional file 1: Figure S1).

## Results

### Study selection

We selected a total of eighteen publications that included 14,846 participants for this meta-analysis. The included studies were selected from 425 potentially related articles (Fig. 1).

### Study characteristics

The detailed characteristics of the involved studies are presented in Table 1. Seven of the included articles were prospective cohort studies [25–31], and 11 were retrospective cohort reports [32–41]. In all of the included publications, the authors tested serum TSH levels before beginning the stimulation protocol. The participant count varied from 98 to 3143 subjects, and the year of publication ranged from 1999 to 2017. Nine of the selected studies reported a high-limit TSH cut-off value. For all included studies, the following results were reported: PR (*n* = 8 using a stricter TSH cut-off value; *n* = 6 using a broader TSH cut-off value), CPR (*n* = 13 using a stricter TSH cut-off value; *n* = 8 using a broader TSH cut-off value), LBR (*n* = 13 using a stricter TSH cut-off value; *n* = 9 using a broader TSH cut-off value), and MR (*n* = 10 using a stricter TSH cut-off value; *n* = 8 using a broader TSH cut-off value) (Table 1).

**Table 1:** The Characteristics of Selected Studies

Author	Year	N	Country	Study design	Assays used	TSH Cut-off	ART details	Outcomes
Coelho	2016	650	Brazil	retrospective	a third-generation assay	2.5:4.0	IVF/ICSI	PR; CPR; LBR; MR
Aghahosseini	2013	816	Iran	retrospective	NS	2.5	IVF	CPR
Mintziori	2014	158	Greece	retrospective	ELISA	2.5	IVF	PR; CPR; LBR; MR
Unuane	2017	3143	Belgium	retrospective	NS	2.5	IUI	PR; LBR; MR
Michalakis	2011	1216	USA	retrospective	NS	2.5;4.0	NS	PR; CPR; LBR; MR
Muller	1999	141	Netherlands	prospective	an immunoluminometric assay	4.5	IVF	PR; LBR; MR
Chai	2014	505	China	retrospective	Access HYPERsensitive hTSH Reagent Pack and Access Free T4 Reagent Pack	2.5;4.5	IVF/ ICSI	CPR; LBR; MR
Baker	2006	146	USA	retrospective	chemiluminometric technology	2.5	IVF	LBR
Karmon	2014	1477	USA	prospective	third-generation assays	2.5	IUI	CPR; LBR
Weghofer	2015	98	USA	retrospective	Electrochemiluminescence immunoassay	2.5	IVF	CPR; LBR
Seungdamrong	2017	1306	USA	prospective	Immulite 2000 system	2.5	IUI	PR; CPR; LBR; MR
Wu	2017	138	China	prospective	NS	2.5; 5	IVF/ ICSI	PR; CPR; LBR; MR
Ba	2013	413	China	prospective	NS	4.2	IVF/ ICSI	PR; CPR; LBR; MR
Zhang	2012	1832	China	retrospective	NS	3.9	IVF/ ICSI	PR; CPR; LBR; MR
Zeng	2014	375	China	prospective	Chemiluminescent immunoassay	2.5;3.5	IVF/ ICSI	CPR; LBR; MR
Gingold	2016	1201	USA	retrospective	NS	2.5;5.0	IVF	PR; CPR; LBR
Reh	2010	1055	USA	retrospective	NS	2.5;4.5	IVF	CPR; LBR; MR
Cai	2017	176	China	prospective	electro- chemiluminescence immunoassays	2.5	IVF	CPR; LBR; MR

*IVF* In vitro fertilization, *ICSI* intracytoplasmatic sperm injection, *IUI* intra-uterine insemination, *ART* assisted reproduction technology, *TSH* thyroid stimulating hormone, *CPR* clinical pregnancy rate, *MR* miscarriage rate, *LBR* live birth rate, *PR* pregnancy rate, *ELISA* enzyme-linked immunosorbent assay

### Synthesis of results and additional analysis

#### Live birth rate

**Fig. 2:**
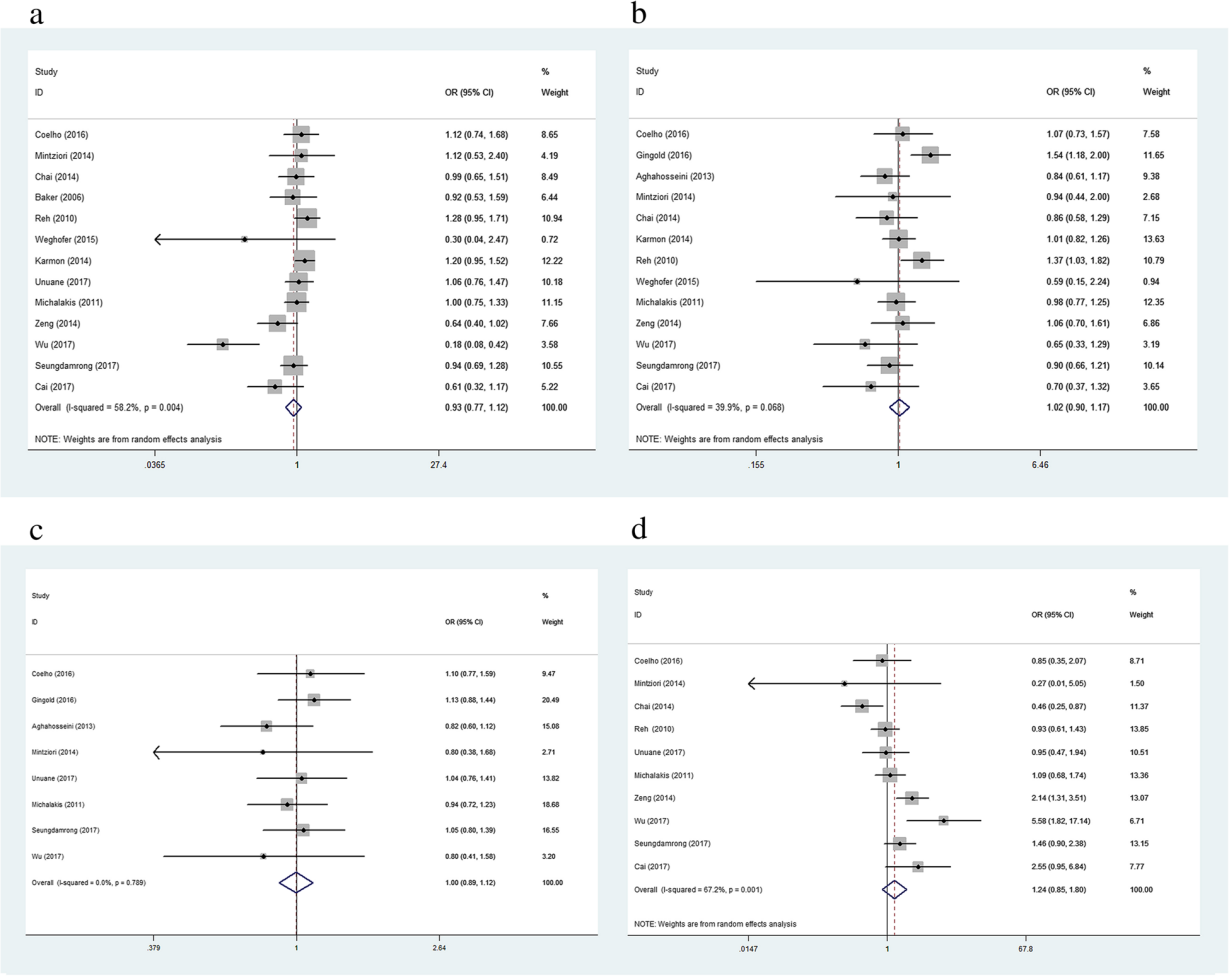
**a-d** Forest plots of studies comparing the number of live birth (**a**); clinical pregnancy (**b**); pregnancy (**c**); miscarriage (**d**) between SCH (TSH cut-off value: 2.5 mIU/L) and euthyroid individuals. The rhombus represents the OR and 95% CI obtained for the combined calculation

Thirteen of the included articles analyzed the LBR as an outcome. Outcomes were pooled from the studies, and the results showed that the LBR was non-significantly lower in women with SCH (TSH cut-off value: 2.5 mIU/L) than in women with normal thyroid function (OR: 0.93; 95% CI (0.77, 1.12), *P* = 0.43). The I^2^ value (58.2%) calculated for these studies indicated high heterogeneity, and we therefore used a random effects model to evaluate the pooled effect estimate (Fig. 2a). A sensitivity analysis demonstrated no difference in the OR when each publication was individually omitted (Additional file 2: Figure S2a). Egger’s test and Begg’s funnel plots (*P* = 0.064) did not indicate the presence of asymmetry for these studies (Additional file 3: Figure S3a).

**Fig. 3:**
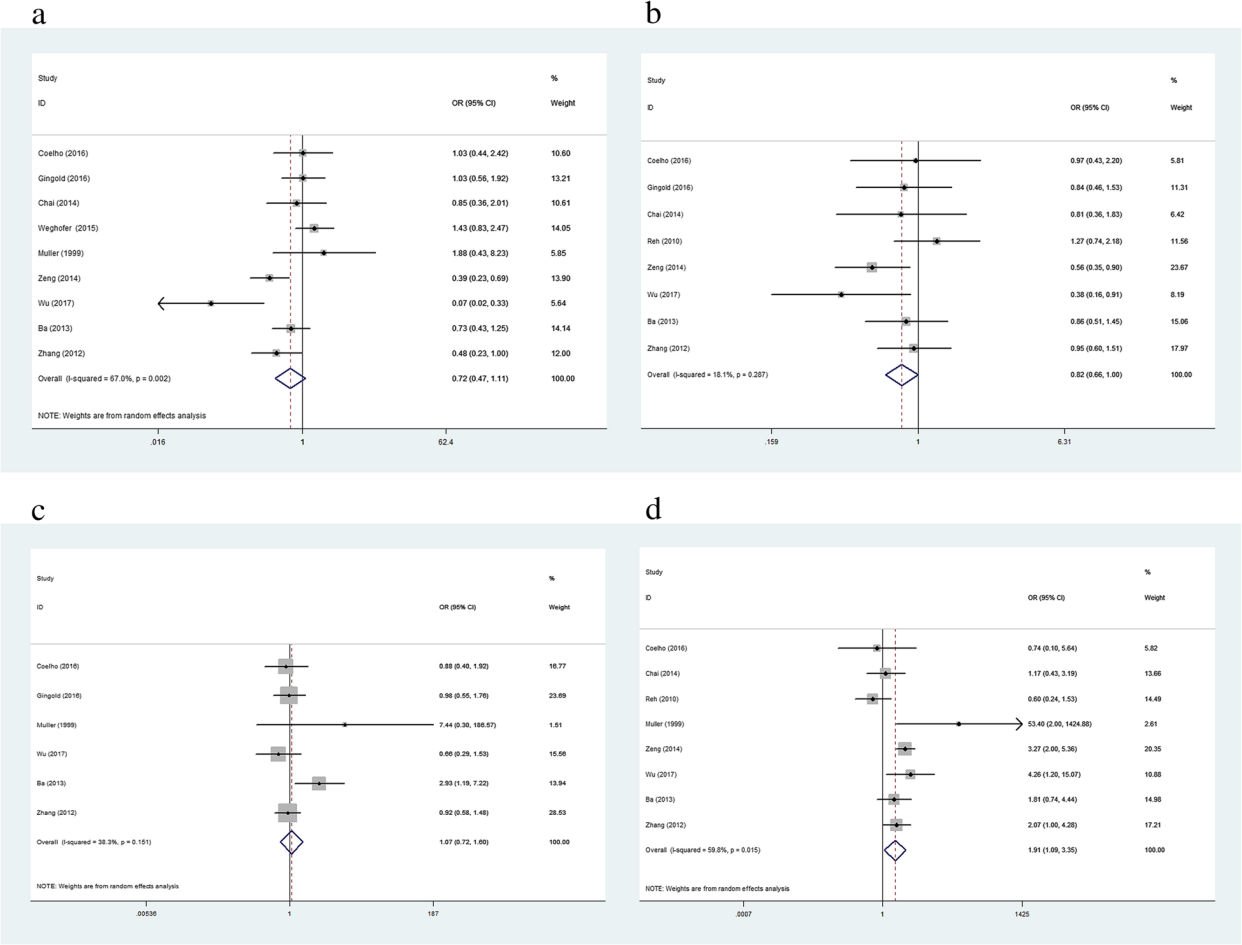
**a-d** Forest plots of studies comparing the number of live birth (**a**); clinical pregnancy (**b**); pregnancy (**c**); miscarriage (**d**) between subclinical SCH (TSH cut-off value is 3.5–5.0 mIU/L) and euthyroid individuals. The rhombus represents the OR and 95% CI obtained for the combined calculation

We also found an insignificant difference in the pooled data for LBR between women with SCH and those with normal thyroid function when a TSH cut-off value of 3.5–5.0 mIU/L was used (OR: 0.72; 95% CI (0.47, 1.11); *P* = 0.14). The I^2^ value (67%) indicated a high degree of heterogeneity. We therefore utilized a random effects model to pool the effect estimate (Fig. 3a). A sensitivity analysis demonstrated no variation in the OR when each trial was individually removed (Additional file 4: Figure S4a). Egger’s test (*P* = 0.57) and Begg’s funnel plots (*P* = 0.92) indicated no asymmetry among these studies (Additional file 5: Figure S5a).

### Secondary outcomes

#### Clinical pregnancy rate

Thirteen of the included articles compared the CPR in participants with and without SCH (TSH cut-off value, 2.5 mIU/L). The meta-analysis indicated no association between SCH and the CPR. Comparison of the CPR between women with SCH and euthyroid women indicated no significant difference (OR: 1.02; 95% CI (0.90, 1.17); *P* = 0.74). The I^2^ value was 39.9%, indicating low heterogeneity. We therefore chose a fixed effects model to pool the effect estimate (Fig. 2b). When each trial was removed, no variation appeared in the direction of the OR (Additional file 2: Figure S2b). Egger’s test (*P* = 0.57) and Begg’s funnel plots (*P* = 0.92) indicated no asymmetry among these studies (Additional file 3: Figure S3b).

We also found an insignificant difference in the pooled data for the CPR between women with SCH and those with normal thyroid function when a cut-off TSH value of 3.5–5.0 mIU/L was used (OR: 0.82; 95% CI (0.66, 1.00); *P* = 0.052). The I^2^ value (18.1%) indicated low heterogeneity. We therefore utilized a fixed effects model to pool the effect estimate (Fig. 3b). A sensitivity analysis demonstrated no variation in the OR when each trial was individually removed (Additional file 4: Figure S4b). Egger’s test (*P* = 0.61) and Begg’s funnel plots (*P* = 0.39) indicated no asymmetry among the studies (Additional file 5: Figure S5b).

#### Pregnancy rate

Nine reports estimated the relationship between SCH (TSH cut-off value, 2.5) and PR. We failed to find a meaningful association between the presence of SCH and the PR (OR: 1.00; 95% CI (0.89, 1.12); *P* = 0.99). The I^2^ value was 0%, indicating an absence of significant heterogeneity (Fig. 2c). A sensitivity analysis showed no variation in the OR when each article was omitted (Additional file 2: Figure S2c). Egger’s test (*P* = 0.21) and Begg’s funnel plots (*P* = 0.27) did not indicate the presence of asymmetry in the involved studies (Additional file 3: Figure S3c).

We also found an insignificant difference in the pooled data for the PR between women with SCH and those with normal thyroid function when a cut-off TSH value of 3.5–5.0 mIU/L was used (OR: 1.07; 95% CI (0.72, 1.60); *P* = 0.74). The I^2^ value (67.2%) indicated high heterogeneity. We therefore utilized a random effects model to pool the effect estimate (Fig. 3c). A sensitivity analysis demonstrated no variation in the OR when each trial was individually removed (Additional file 4: Figure S4c). Egger’s test (*P* = 0.27) and Begg’s funnel plots (*P* = 0.45) indicated no asymmetry among the studies (Additional file 5: Figure S5c).

#### Miscarriage rate

A total of ten publications assessed the association between SCH and miscarriage. A meta-analysis of these 7 reports indicated an insignificant difference in the risk of miscarriage between euthyroid women and SCH patients (OR: 1.24; 95% CI (0.85, 1.80); *P* = 0.26). The I^2^ value was 67.2%, indicating high heterogeneity. We therefore chose a random effects model to pool the effect estimate (Fig. 2d). A sensitivity analysis showed no variation in the OR when each article was individually removed (Additional file 2: Figure S2d). Egger’s test (*P* = 0.85) and Begg’s funnel plots (*P* = 0.59) indicated no asymmetry in the studies (Additional file 3: Figure S3d).

We also found a significant difference in the pooled data for the MR between women with SCH and those with normal thyroid function when a TSH cut-off value of 3.5–5.0 mIU/L was used (OR: 1.91; 95% CI (1.09, 3.35); *P* = 0.02). The I^2^ value (59.8%) indicated high heterogeneity. We therefore utilized a random effects model to pool the effect estimate (Fig. 3d). A sensitivity analysis demonstrated no variation in the OR when each trial was individually removed (Additional file 4: Figure S4d). Egger’s test (*P* = 0.83) and Begg’s funnel plots (*P* = 0.90) indicated no asymmetry among these studies (Additional file 5: Figure S5d).

#### Quality assessment and risk of bias

The quality assessment of the studies included in the meta-analysis is shown in Table 2. An overview and summary of probable risks of bias across all included studies is showed in Table 3. Selection bias was rated as high risk across five researches. In terms of potential performance bias, five studies were found to be of high risk. Detection bias was potentially rated as high risk in eight studies. Only four studies potentially posed high risk for potential attrition bias. Reporting bias was potentially rated as high risk across five studies. Other sources of biases included the sampling bias and measurement bias. Sampling bias was judged as high risk in one study. Furthermore, measurement bias was rated as high risk in ten studies.

**Table 2:** Quality of the studies on the Newcastle-Ottawa scale

Study	Selection	Comparability	Exposure/Outcome	Total Stars
Coelho 2016	*****	*	**	8
Aghahosseini 2013	****	**	**	8
Mintziori 2014	****	**	*	7
Unuane 2017	***	**	**	7
Michalakis 2011	****	**	*	7
Muller 1999	***	**	**	7
Chai 2014	****	*	**	7
Weghofer 2015	****	**	*	7
Baker 2006	***	**	**	7
Karmon 2014	****	**	**	8
Cai 2017	****	**	**	8
Seungdamrong 2017	****	**	**	8
Zhang 2012	*****	**	*	8
Ba 2013	***	**	**	7
Reh 2010	***	**	**	7
Zeng 2014	*****	*	**	8
Gingold 2016	****	**	*	7
Wu 2017	****	**	*	7

* means 1 score

**Table 3:** Assessment of risk of bias in individual studies

Study	Selection bias	Performance bias	Detection bias	Attrition bias	Reporting bias	Other bias	
						Sampling bias	Measurement bias
Coelho 2016	–	+	–	–	–	?	–
Aghahosseini 2013	–	–	?	–	+	–	–
Mintziori 2014	+	–	+	–	–	–	+
Unuane 2017	+	–	–	–	–	+	+
Michalakis 2011	+	?	+	–	–	?	+
Muller 1999	–	+	–	+	–	?	–
Chai 2014	+	+	–	–	–	–	+
Weghofer 2015	–	?	+	+	–	?	+
Baker 2006	?	–	+	–	+	–	–
Karmon 2014	–	?	–	–	+	–	–
Cai 2017	–	–	?	–	–	?	+
Seungdamrong 2017	–	–	–	+	–	–	+
Zhang 2012	–	+	–	+	–	–	+
Ba 2013	+	–	?	–	–	–	–
Reh 2010	–	+	+	–	+	?	+
Zeng 2014	?	–	+	–	–	?	–
Gingold 2016	?	–	+	–	–	–	+
Wu 2017	–	?	+	–	+	–	–

+ high risk of bias; − low risk of bias;? unclear risk of bias

## Discussion

This meta-analysis was specifically aimed at evaluating the associations between preconception maternal TSH levels and ART outcomes. We found that a stricter TSH cut-off value did not seem to influence ART outcomes including the LBR, PR, CPR, and MR. Furthermore, using a broader TSH cut-off value resulted in a significant difference in poor ART outcomes including the MR.

Many previous studies have researched the association between SCH during conception and maternal outcomes, but their conclusions have been inconsistent. In 2016, a meta-analysis focused on thyroid dysfunction indicated that SCH during pregnancy is related to multiple poor neonatal and maternal outcomes, especially abortion [9]. However, the studies evaluated in that analysis had enrolled participants who were normal fertile woman. However, we should not ignore the fact that more and more infertile patients are undergoing ART treatment. Recently, many articles have focused on the relationship between preconception SCH in infertile patients and multiple ART outcomes. However, their conclusions have been conflicting. Nevertheless, this information is very important because most reproductive endocrinologists routinely perform preconception tests for serum TSH levels as a part of an elementary infertility workup.

Moreover, subclinical thyroid function disorder is a focus of studies evaluating patients undergoing ovarian stimulation because it has been suggested that these patients experience a decrease in thyroxin levels and an increase in TSH levels during ovulation induction.

Due to the increased subsequent risk of hypothyroidism in participants with a TSH level above 2.0 mIU/L (e.g., as demonstrated in the Whickham survey [42]), the NACB guidelines suggest that 2.5 mIU/L should be regarded as the upper limit for a normal TSH reference range [43]. In patients previously diagnosed with hypothyroidism, the Endocrine Society (TES) practice guidelines suggest that preconception TSH values should be under 2.5 mIU/ mL [44]. The ATA and the American Association of Clinical Endocrinologists co-sponsored guidelines for the treatment of hypothyroidism in patients attempting to conceive [19]. Their proposal reinforces the notion that TSH levels should be maintained at lower than 2.5 mIU/L in women with hypothyroidism, including those with overt hypothyroidism and SCH before pregnancy. Moreover, there is a lack of consistency regarding what cut-off value for serum TSH levels should be used to define SCH in individuals undergoing ART. Several studies have relied on the use of a recommended basal cut-off value of TSH < 2.5 mIU/l in individuals termed “desirable” for conception. However, we cannot ignore that some prospective and retrospective studies found no difference in IVF outcomes between women with serum TSH < 2.5 mU/L and those with mild TSH elevations, defined as a TSH level between 2.5 and 5 mU/L. The results of these studies were inconsistent with our outcomes. The underlying reason remains uncertain. One of the probable reasons is the distribution of TSH levels in infertile women. Braverman et al. found that the distribution of TSH levels in infertile individuals presented a left skew with a long tail [20]. The argument for decreasing the upper TSH reference range presumes that it conforms to a Gaussian distribution in nature. However, studies have found that in some euthyroid outliers, such as patients recovering from nonthyroidal illness, measurements that include bioinactive TSH isoforms or receptor gene polymorphisms as well as occult autoimmune thyroid dysfunction show that the upper tail of the distribution is skewed [45, 46]. Additionally, TSH levels can be influenced by strenuous exercise, the timing of phlebotomy and sleep deprivation [47]. Previous publications have also indicated that the distribution of TSH levels gradually shifts toward higher values with age [48]. Hence, TSH concentrations could represent an epiphenomenon in which the above-mentioned factors account for its influence on pregnancy outcomes.

When a broader TSH cut-off value was used, we found a significant difference in the MR between SCH patients and normal women. This outcome is consistent with some random clinical trials (RCTs). These trials demonstrated that treatment of SCH patients who were defined using a broader TSH cut-off value appeared beneficial. An RCT was conducted in patients aged 20–40 years old with SCH (serum TSH > 4.5 mU/L, normal fT4) who were undergoing IVF [49]. A total of 64 participants were randomized for supplementation with LT4 (to maintain TSH levels < 2.5 mU/L) vs. placebo. The treated patients had MRs, higher CPRs, and higher LBRs. Another RCT randomized 64 infertile women with SCH (TSH > 4.2 mU/L, normal fT4) to a treatment group receiving 50 mcg/day LT4 vs. a placebo group [50]. Similar to the above trial, the study showed higher PRs, lower MRs, and higher LBRs in the treatment group than in the control group. Taken together, these data suggest that SCH likely impacts ART in a dose-dependent manner, and the impact worsens as TSH concentrations increase. Hence, in 2017, the ATA guidelines recommended that women with SCH undergoing IVF or ICSI should be treated with LT4 with a goal of maintaining a TSH concentration < 2.5 mU/L. It is worth noting that this recommendation is focused on women who are undergoing ART. However, the effect of SCH before ART on ART outcomes remains uncertain. Our results suggest that applying a broader TSH cut-off value during the preconception period may improve ART outcomes such as the pregnancy loss rate.

In addition, we found that an increasing number of clinicians are uncertain regarding whether to perform preconception thyroid function tests, especially for women seeking ART [51]. Routine preconception testing for all women is not recommended by any relevant professional body (ATA, ACOG, RCOG) primarily because there is no high-level evidence to support its use. Our search also provided high-level evidence regarding the necessity of routine thyroid function tests before conception in women seeking to ART. We found that women with SCH defined using the broader TSH cut-off value before conception could have poor ART outcomes, such a higher MR.

In contrast to our findings, a recent prospective trial published by Negro et al. [7] included 4123 thyroid antibody–negative subjects and found that the risk of miscarriage was higher in women with TSH values between 2.5 and 5.0 mIU/L at < 11 weeks of pregnancy. The reasons for this discrepancy may include the following. For example, the subjects were individuals with spontaneous conceptions who were already in the first trimester and did not include infertile women. Therefore, TSH levels were tested only in subjects who were already pregnant, whereas in our study, all TSH concentrations were measured before conception. This difference could be partially responsible for the difference in the effect in that if TSH values decreases during early pregnancy, as suggested in a previous study [52], then individuals with TSH values above 2.5 mIU/L during the first trimester might have had higher TSH levels before pregnancy. In addition, ovarian stimulation seems to influence TSH concentrations [24, 53].

### Strengths and limitations of this meta-analysis

There are several limitations to this meta-analysis. First, the sizes of the populations included in many of the original studies and the total number of patients were small. Second, because some original articles were retrospective, it is probable that other factors may account for the differences observed in outcomes. In retrospective cohort studies, it is feasible that there will be selection bias, problems with the quality of the original publications, publication bias, confounding and heterogeneity. Third, TPO, an important confounding factor, could not be eliminated in several of the original papers because of limitations associated with the studies. In spite of these limitations, the current meta-analysis has some strengths. Our methodology was rigorous, and the outcomes achieved after the results of these trials were pooled were more credible. We also comprehensively compared the CPR, PR and MR as secondary outcomes between normal women and SCH patients to explore the influence of preconception SCH on ART outcomes. Furthermore, we analyzed the data using two different cut-off values for TSH levels to explore the association between SCH and adverse ART outcomes. The results suggested a new diagnosis standard to define SCH in woman seeking ART.

## Conclusions

Our data indicate no difference in ART outcomes when a TSH cut-off value of 2.5 mIU/L is used. However, using a broader cut-off value of TSH, we found a higher MR in SCH patients seeking ART than in the control group. We suggest that a thyroid function test should be a routine examination in women seeking ART.

## Additional files

Additional file 1: **Figure S1.** PRISMA Checklist. (EPS 538 kb)

Additional file 2: **Figure S2.** a-d Sensitivity analysis of the studies included in the meta- analysis. The figure (a-d) shows the OR obtained by combined analysis of the remaining studies after the successive exclusion of each study individually. The excluded study is listed on the left, and the corresponding horizontal lines indicate the OR and CI obtained by re-calculation after its exclusion. The CI for the overall meta-analysis of the studies is indicated by two vertical lines. (TIF 746 kb)

Additional file 3: **Figure S3.** a-d Begg’s funnel plot for publication bias analysis. Each point represents a separate study. (TIF 347 kb)

Additional file 4: **Figure S4.** a-d Sensitivity analysis of the studies included in the meta- analysis. The figure (a-d) shows the OR obtained by combined analysis of the remaining studies after the successive exclusion of each study individually. The excluded study is listed on the left, and the corresponding horizontal lines indicate the OR and CI obtained by re-calculation after its exclusion. The CI for the overall meta-analysis of the studies is indicated by two vertical lines. (TIF 587 kb)

Additional file 5: **Figure S5.** a-d Begg’s funnel plot for publication bias analysis. Each point represents a separate study. (TIF 316 kb)

## Abbreviations

AACC: American Association for Clinical Chemistry
ART: Assisted reproductive technology
ASRM: American Society for Reproductive Medicine
ATA: American Thyroid Association
CI: Confidence interval
CPR: Clinical pregnancy rate
ESHRE: European Society of Human Reproduction and Embryology
ICSI: Intra-cytoplasmic sperm injection
IUI: Intrauterine insemination
IVF: In vitro fertilization
LBR: Live birth rate
MR: Miscarriage rate
NACB: National Association of Clinical Biochemistry
OH: Overt hypothyroidism
OR: Odds ratio
PR: Pregnancy rate
SCH: Subclinical hypothyroidism
T4: Thyroxine
TES: The Endocrine Society
TSH: Thyroid-stimulating hormone
