# Supplementary figures and images for: Meta-analysis of ART outcomes in women with different preconception TSH levels
Source: Reprod Biol Endocrinol. 2018 Nov 5;16:111. doi: 10.1186/s12958-018-0424-0 (PMC6219175; doi:10.1186/s12958-018-0424-0)

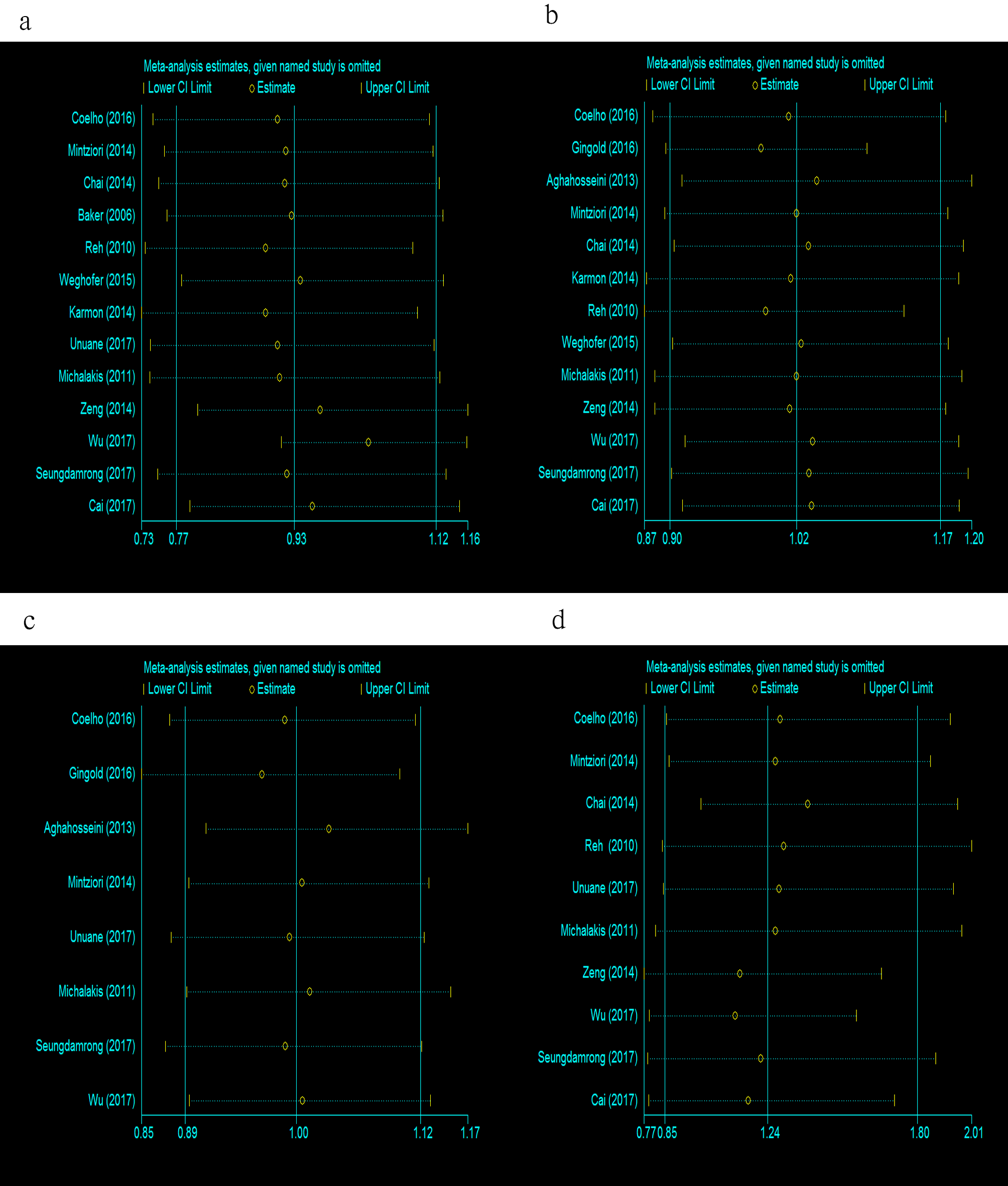

Supplement: Supplementary file 2 — Figure S2. a-d Sensitivity analysis of the studies included in the meta- analysis. The figure (a-d) shows the OR obtained by combined analysis of the remaining studies after the successive exclusion of each study individually. The excluded study is listed on the left, and the corresponding horizontal lines indicate the OR and CI obtained by re-calculation after its exclusion. The CI for the overall meta-analysis of the studies is indicated by two vertical lines. (TIF 746 kb) [file 12958_2018_424_MOESM2_ESM.tif]

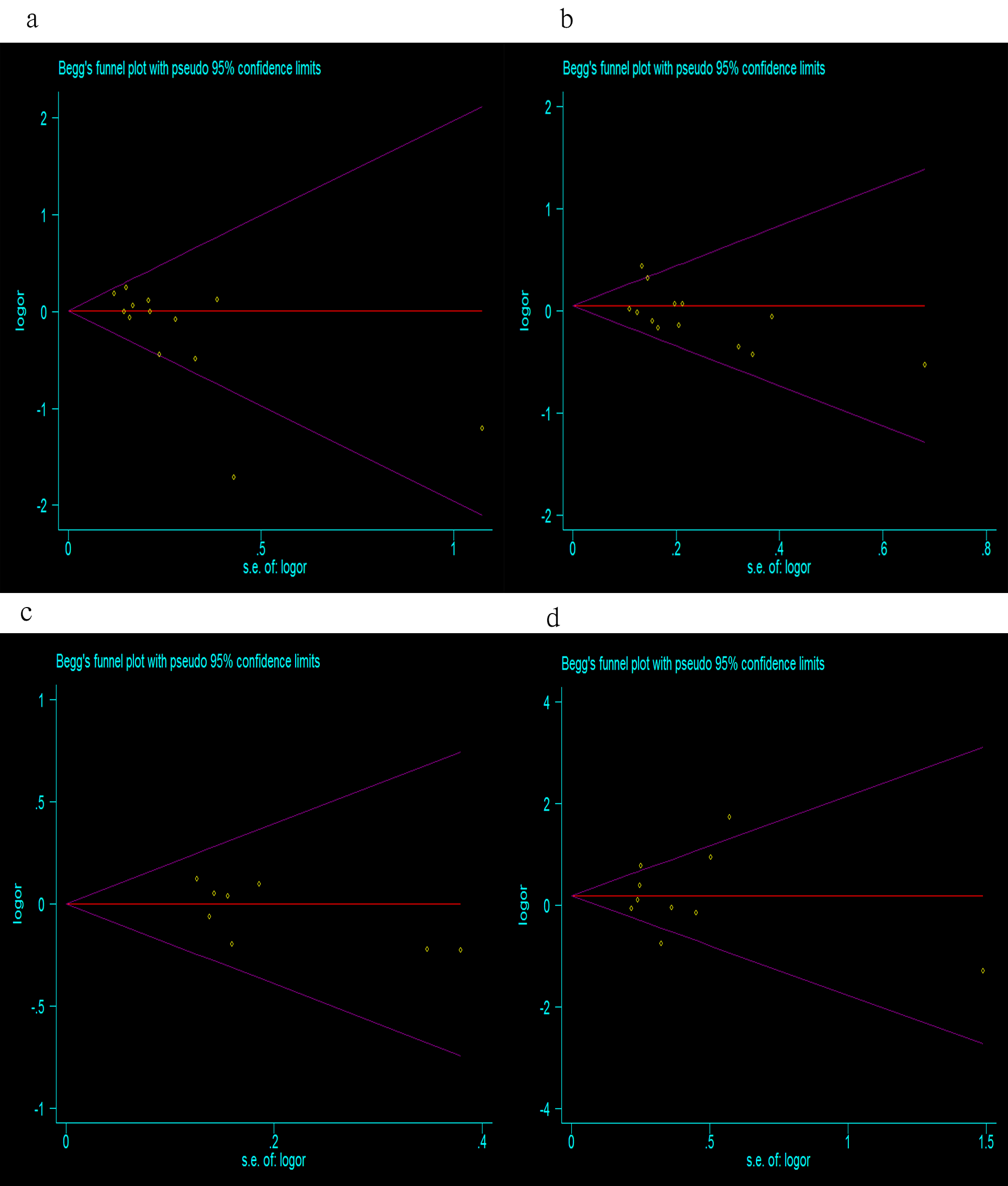

Supplement: Supplementary file 3 — Figure S3. a-d Begg’s funnel plot for publication bias analysis. Each point represents a separate study. (TIF 347 kb) [file 12958_2018_424_MOESM3_ESM.tif]

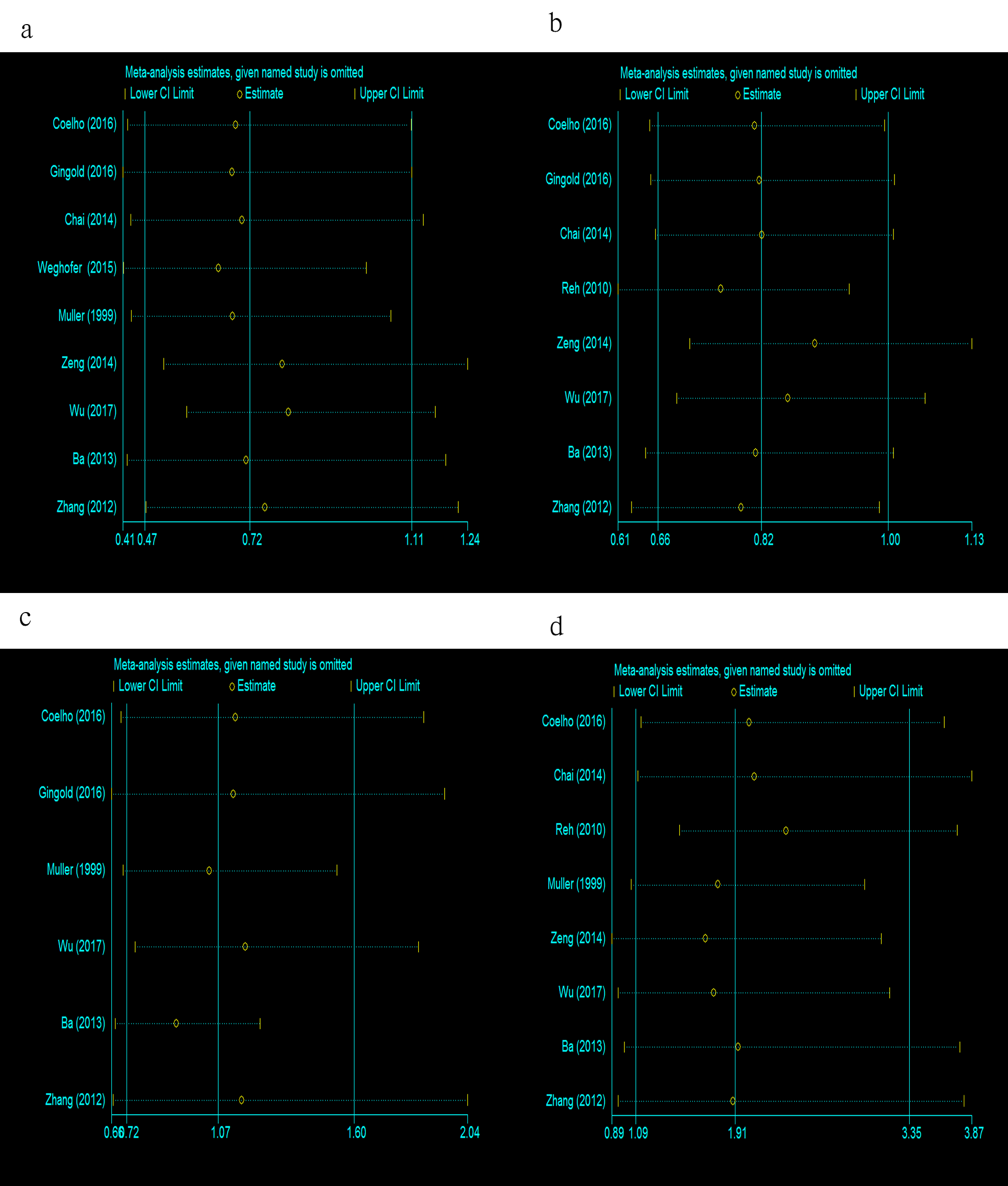

Supplement: Supplementary file 4 — Figure S4. a-d Sensitivity analysis of the studies included in the meta- analysis. The figure (a-d) shows the OR obtained by combined analysis of the remaining studies after the successive exclusion of each study individually. The excluded study is listed on the left, and the corresponding horizontal lines indicate the OR and CI obtained by re-calculation after its exclusion. The CI for the overall meta-analysis of the studies is indicated by two vertical lines. (TIF 587 kb) [file 12958_2018_424_MOESM4_ESM.tif]

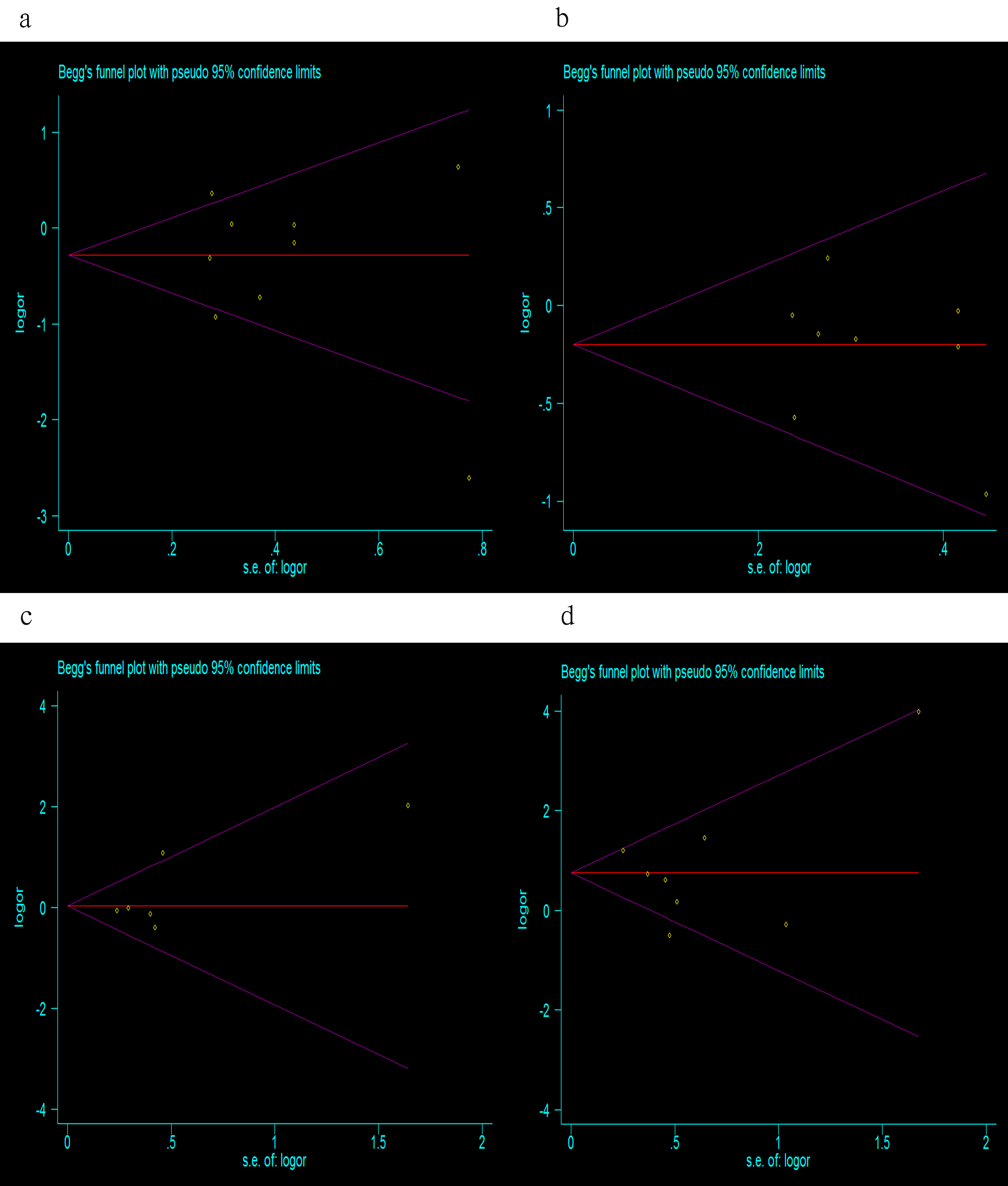

Supplement: Supplementary file 5 — Figure S5. a-d Begg’s funnel plot for publication bias analysis. Each point represents a separate study. (TIF 316 kb) [file 12958_2018_424_MOESM5_ESM.tif]
